# Comparison between Amblyopia Treatment with Glasses Only and Combination of Glasses and Open-Type Binocular “Occlu-Pad” Device

**DOI:** 10.1155/2018/2459696

**Published:** 2018-02-19

**Authors:** Yo Iwata, Tomoya Handa, Hitoshi Ishikawa, Toshiaki Goseki, Nobuyuki Shoji

**Affiliations:** ^1^Doctor's Program of Medical Science, Kitasato University Graduate School, 1-15-1 Kitasato, Sagamihara 252-0374, Japan; ^2^Department of Rehabilitation, Orthoptics and Visual Science Course, School of Allied Health Sciences, Kitasato University, 1-15-1 Kitasato, Sagamihara 252-0373, Japan; ^3^Department of Ophthalmology, School of Medicine, Kitasato University, 1-15-1 Kitasato, Sagamihara 252-0374, Japan

## Abstract

We evaluated amblyopia treatment, comparing training with glasses only and training with glasses and the Occlu-pad, a binocular open-type amblyopia training device. Forty-six children (4.8 ± 1.1 years) diagnosed with anisometropic amblyopia, all wearing complete correction glasses, were treated either with glasses only, or with glasses in combination with the Occlu-pad (training time: 2 days a week, 30 minutes per day). We compared visual acuity scores at 3 and 6 months after treatment had started, and examined the compliance rate for the Occlu-pad training. Three months as well as 6 months after amblyopia treatment started, the “Occlu-pad treatment group” showed significantly improved visual acuity, compared to the “Glasses treatment group” (at both 3 and 6 months: *p* < 0.0001). The compliance rate for using the Occlu-pad was 88.4 ± 18.7% after 3 months and 69.6 ± 19.5%, after 6 months. There was no significant correlation between the training time using the Occlu-pad and improvement in visual acuity (3 months: *p* = 0.97; 6 months: *p* = 0.55). The compliance rate for months 4 to 6 was significantly lower than that for months 1 to 3 (*p* = 0.003). Amblyopia treatment using the Occlu-pad device in combination with glasses led to a better effect than treatment with glasses alone.

## 1. Introduction

Amblyopia is reported to be present in about 1–5% of children [1–4], and it affects children's development, their academic work, and various aspects of their social life. It has also been reported that people who have amblyopia in one eye are about twice as likely to develop vision disorders in both eyes when reaching a certain age [5]. The treatment of amblyopia is therefore extremely important. In conventional clinical ophthalmology, the gold standard for amblyopia treatment is wearing complete refractive correction glasses. In addition, occlusion therapy by using an eyepatch on the healthy eye is used for more aggressive treatment. Occlusion therapy is important because there are reports that approximately 70% of patients do not regain good visual acuity by only wearing eyeglasses [6]. However, occlusion therapy has side effects, such as occlusion amblyopia, skin rashes, and mental distress [7], and compliance is extremely poor [8, 9]. In recent years, due to the development of image technology, a variety of amblyopia treatment devices that present the visual target to only the amblyopic eye under open binoculars have been developed [10–13].

It has been suggested that the effect of amblyopia treatment by using open-type binocular devices may be better than that of occlusion therapy by using an eyepatch [11, 12]. In Japan, a device named “Occlu-pad” (outside Japan, it is called “Occlu-tab”), which is a tablet terminal subjected to special processing, is used in clinical ophthalmology [13]. The Occlu-pad uses white-screen technology to present target images selectively to one eye under open binoculars. The white-screen technology involves peeling off the polarizing film layer of a liquid crystal panel, and, by attaching this peeled film to glasses, viewing videos is only possible when the subject is wearing the polarized glasses. For example, if the film is attached to the right-eye lens of glasses, the subject can view the image only in the right eye. The Occlu-pad has succeeded in producing good amblyopia training results for anisometropic amblyopia [13]. However, a comparison between amblyopia training effects of glasses only and amblyopia training effects of glasses in combination with the Occlu-pad has not been undertaken so far. In addition, it is difficult to lend or sell the device to all patients with amblyopia, because, unlike eye patches, open-type binocular amblyopia treatment devices including the Occlu-pad are special electronic equipment. Therefore, in this study, we investigated amblyopia training effects using the Occlu-pad in a hospital setting, that is, in patients who visited our clinic to receive treatment for amblyopia.

## 2. Methods and Materials

The study conformed to the tenets of the Declaration of Helsinki and was approved by the Kitasato University Human Sciences Ethics Committee (B-16-85). All procedures were carried out in accordance with approved guidelines. Informed consent was obtained from all subjects after the nature and possible consequences of the study had been explained to them.

We included patients who visited the hospital, were diagnosed with anisometropic amblyopia (refraction difference of both eyes 2D or more; highest visual acuity of amblyopic eyes 0.1 (LogMAR) or less), were between 3 and 8 years of age, and revisited the hospital at 3 and 6 months after the start of the amblyopia training.

Patients with an amblyopia treatment history or with strabismus, patients in whom it was difficult to perform visual acuity and refraction tests, patients with an astigmatism of 1.50 D or greater, and patients who underwent occlusion therapy using an eye patch were excluded.

This study was a randomized controlled trial. Randomization was performed using the permuted block method, with a block size of 2. The permuted block method was performed using random numbers in Excel. The report of treatment with Occlu-pad alone is only 3 cases [13]. For visual acuity differences (LogMAR) between the two groups, 0.1 ± 0.1 was estimated. For *α* = 0.05 and 1 − *β* of 0.90, the required sample size was 22 children per group. To account for an anticipated 5% dropout rate, we enrolled 46 children (23 per group).

The subjects were 46 children (mean age ± standard deviation: 4.8 ± 1.1 years, range 3–7 years) diagnosed with anisometropic amblyopia. All patients were wearing complete correction glasses fitted under cycloplegic refraction.

**Figure 1:**
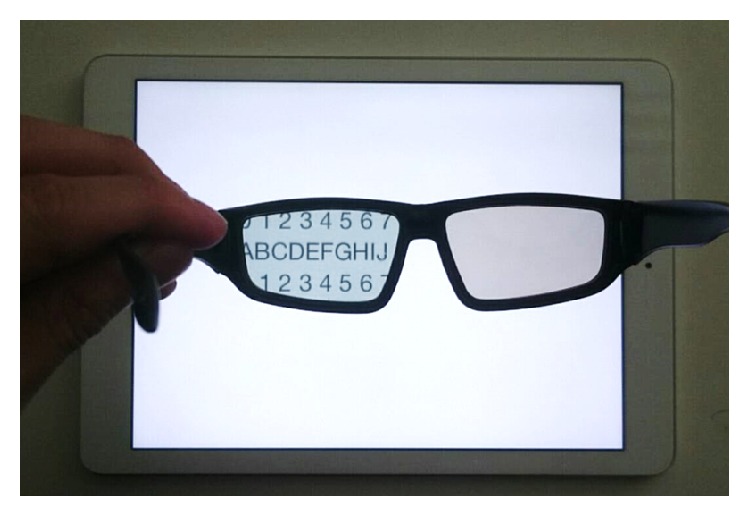
Appearance of the Occlu-pad. Only the left eye can see the image of the tablet terminal, while the right eye cannot.

Twenty-three of the children were treated for amblyopia with only glasses (“Glasses treatment group”: 4.9 ± 1.1 years of age), while the other 23 patients were treated with glasses in combination with the Occlu-pad (“Occlu-pad treatment group”: 4.8 ± 1.2 years). The Occlu-pad can present target images on a tablet terminal to only the amblyopic eye under open binoculars (Figure 1). The doctor instructed the patients' parents to perform training with the Occlu-pad for 2 days a week (30 minutes per day). During training using the Occlu-pad, the patient played an arbitrary game requiring eye-hand coordination. The orthoptists and parents confirmed whether the child was training properly. Visual acuity (LogMAR) and the refractive difference between the healthy and amblyopic eye in the Glasses treatment group and the Occlu-pad treatment group were 0.24 ± 0.08 (Glasses group) versus 0.25 ± 0.09 and 2.88 ± 0.57 D (Glasses group) versus 3.11 ± 0.60 D, respectively. We compared visual acuity scores at 3 months and at 6 months after amblyopia treatment started. For the visual acuity test, the Landolt ring chart was used. We also examined the compliance rate in the Occlu-pad treatment group. The Mann–Whitney* U* test was used for comparisons between the Glasses treatment group and the Occlu-pad treatment group. Wilcoxon's signed-rank test was used to assess the compliance rate in the Occlu-pad group. The Kendall rank correlation coefficient was used for correlations of improvement in visual acuity and compliance in the Occlu-pad group. The normality of the data has been confirmed by a Kolmogorov-Smirnov test. A *p* < 0.05 was considered statistically significant.

## 3. Results

Measures of visual acuity (LogMAR) in the Glasses treatment group and the Occlu-pad treatment group before training, 3 months after starting the training, and 6 months after starting the training are shown in Table 1.

**Figure 2:**
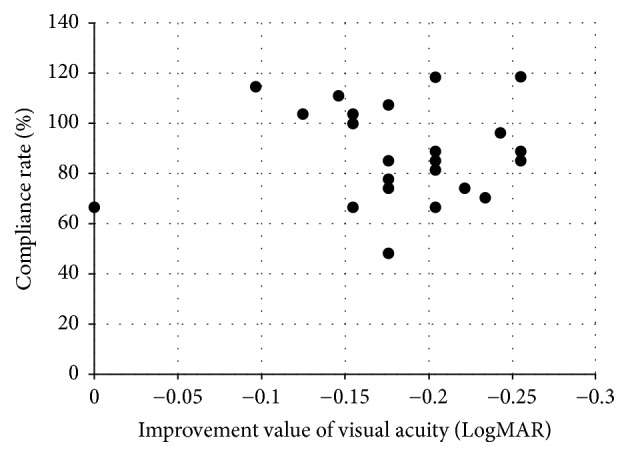
Correlation between improvement in visual acuity measures and compliance rate 3 months after starting amblyopia training.

**Figure 3:**
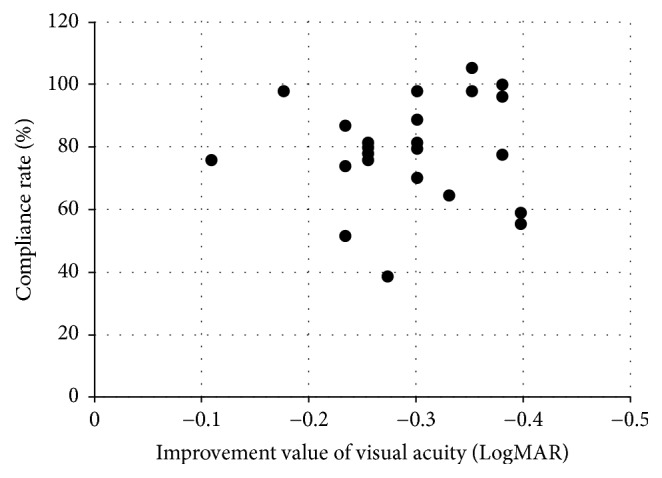
Correlation between improvement in visual acuity measures and compliance rate 4 months to 6 months after starting amblyopia training.

There was no significant difference in age, refractive difference between healthy and amblyopic eye, or visual acuity before starting amblyopia treatment between the two groups (Glasses treatment group and Occlu-pad treatment group) (*p* = 0.65, *p* = 0.20, and *p* = 0.65, resp.). Three months after the treatment started, the corrected visual acuity in the Glasses treatment group and the Occlu-pad treatment group was 0.14 ± 0.08 and 0.06 ± 0.09, respectively. Participants in the Occlu-pad treatment group improved their visual acuity significantly, compared to those in the Glasses treatment group (*p* < 0.0001). The compliance rate (conducted training time/instructed training time) for using the Occlu-pad in the Occlu-pad treatment group was 88.4 ± 18.7%. There was no significant correlation between the training time (compliance rate) using the Occlu-pad and improvement in visual acuity (*p* = 0.97) (Figure 2). Six months after amblyopia treatment started, the corrected visual acuity in the Glasses treatment group and the Occlu-pad treatment group was 0.05 ± 0.09 and −0.05 ± 0.08, respectively. Participants in the Occlu-pad treatment group improved their visual acuity significantly, compared to the Glasses treatment group (*p* < 0.0001). The compliance rate for using the Occlu-pad in the Occlu-pad treatment group was 69.6 ± 19.5%. There was no significant correlation between the training time (compliance rate) using the Occlu-pad and improvement in visual acuity (*p* = 0.55) (Figure 3). The compliance rate during months 4 to 6 was significantly lower than that during months 1 to 3 (*p* = 0.003).

## 4. Discussion

In this study, a good amblyopia training effect was obtained by using the Occlu-pad, which is an open-type binocular amblyopia training device.

Training with the Occlu-pad in this study was restricted to a very short time (2 days a week, 30 minutes per day), because the training was performed during hospital visits only. However, a more effective amblyopia training effect was obtained using the Occlu-pad in combination with wearing glasses, compared with glasses only. As for training under occlusion therapy using an eye patch, it has been reported that there is no significant difference between full-time occlusion and a 6-hour occlusion per day [14] and that there is no significant difference between a 6-hour occlusion per day and a 2-hour occlusion per day [15]. It therefore seems reasonable to assume that amblyopia training is successful even when restricted to a short time. Furthermore, in this study, there was no significant correlation between training time with the Occlu-pad and improvement in visual acuity. This suggests that prolonging the training time may not necessarily lead to a more pronounced amblyopia training effect.

The compliance rate for occlusion therapy using an eye patch is low [8], which is problematic. The compliance rate for using the Occlu-pad in this study was however very high. In the past, it has been reported that there is a positive correlation between frequency of hospital visits and compliance rate during amblyopia treatment [8]. By repeating training sessions during hospital visits, patients were able to raise their awareness for the training, which presumably led to the good compliance rate. In addition, we consider that it is a great merit that training by hospital visit can fully grasp the training time. However, since amblyopia treatment using the Occlu-pad in a hospital setting may be a burden to patients and parents especially for people living far from a hospital, it is necessary to pay attention to this when doing this method.

It has been suggested that open-type binocular treatment can obtain good amblyopia training results, compared to occlusion therapy with an eye patch [11, 12]. This is because occlusion therapy with an eye patch forces the patient to use the amblyopic eye, but during open-type binocular treatment with the Occlu-pad, the amblyopic eye needs to be used spontaneously. Therefore, we assume that open-type binocular therapy is more effective than occlusion therapy due to the elimination of suppression. However, this study did not compare Occlu-pad treatment with occlusion therapy using an eye patch. Further studies are thus needed in the future to compare these two treatment strategies.

## 5. Conclusions

This study compared amblyopia treatment training with glasses only to training with glasses and the Occlu-pad device, an open-type binocular amblyopia training device, and found a significant improvement in visual acuity for the Occlu-pad training group. Since compliance rates for the device were also found to be very high, this combination treatment offers a promising alternative to conventional treatment of amblyopia.

## Figures and Tables

**Table 1:** Measurements of visual acuity (LogMAR) in the Glasses treatment group and the Occlu-pad treatment group, before training, 3 months after starting the training, and 6 months after starting the training.

	Before training	At 3 months	At 6 months
Glasses treatment group	0.24 ± 0.08	0.14 ± 0.08	0.05 ± 0.09
Occlu-pad treatment group	0.25 ± 0.09	0.06 ± 0.09	−0.05 ± 0.08
